# Improving the Energy Storage Performance in Bi_0.5_Na_0.5_TiO_3_-Based Ceramics by Combining Relaxor and Antiferroelectric Properties

**DOI:** 10.3390/ma17205044

**Published:** 2024-10-15

**Authors:** Srinivas Pattipaka, Yeseul Lim, Yundong Jeong, Mahesh Peddigari, Yuho Min, Jae Won Jeong, Jongmoon Jang, Sung-Dae Kim, Geon-Tae Hwang

**Affiliations:** 1 Department of Materials Science and Engineering, Pukyong National University, 45, Yongso-ro, Nam-Gu, Busan 48513, Republic of Korea; cnuphy444@gmail.com (S.P.); imyeseul21@pukyong.ac.kr (Y.L.); yd3998@pukyong.ac.kr (Y.J.); sdkim@pknu.ac.kr (S.-D.K.); 2 Department of Physics, Indian Institute of Technology Hyderabad, Kandi 502284, Telangana, India; mahesh.p@phy.iith.ac.in; 3 School of Materials Science and Engineering, Kyungpook National University, 80 Daehak-ro, Buk-gu, Daegu 41566, Republic of Korea; yuhomin@knu.ac.kr; 4 Korea Institute of Materials Science (KIMS), 797 Changwondaero, Seongsan-gu, Changwon 51508, Republic of Korea; jeongjw1204@kims.re.kr; 5 Department of Electronic Engineering, Yeungnam University, Gyeongsan 38541, Republic of Korea; jongmoon@yu.ac.kr

**Keywords:** ceramic capacitors, lead-free ceramics, relaxor and antiferroelectrics, energy storage density, energy efficiency

## Abstract

Ceramic capacitors have received great attention for use in pulse power systems owing to their ultra-fast charge–discharge rate, good temperature stability, and excellent fatigue resistance. However, the low energy storage density and low breakdown strength (BDS) of ceramic capacitors limit the practical applications of energy storage technologies. In this work, we present a series of relaxor ferroelectric ceramics (1−*x*) [0.94 Bi_0.5_Na_0.5_TiO_3_ –0.06BaTiO_3_]– *x* Sr_0.7_Bi_0.2_TiO_3_ (1-*x* BNT-BT- *x* SBT; *x* = 0, 0.20, 0.225, 0.25, 0.275 and 0.30) with improved energy storage performances by combining relaxor and antiferroelectric properties. XRD, Raman spectra, and SEM characterizations of BNT-BT-SBT ceramics revealed a rhombohedral–tetragonal phase, highly dynamic polar nanoregions, and a reduction in grain size with a homogeneous and dense microstructure, respectively. A high dielectric constant of 1654 at 1 kHz and low remnant polarization of 1.39 µC/cm^2^ were obtained with the addition of SBT for *x* = 0.275; these are beneficial for improving energy storage performance. The diffuse phase transition of these ceramics displays relaxor behavior, which is improved with SBT and confirmed by modified the Curie–Weiss law. The combining relaxor and antiferroelectric properties with fine grain size by the incorporation of SBT enables an enhanced maximum polarization of a minimized *P-E* loop, leading to an improved BDS. As a result, a high recoverable energy density *W_rec_* of 1.02 J/cm^3^ and a high energy efficiency *η* of 75.98% at 89 kV/cm were achieved for an optimum composition of 0.725 [0.94BNT-0.06BT]-0.275 SBT. These results demonstrate that BNT-based relaxor ferroelectric ceramics are good candidates for next-generation ceramic capacitors and offer a potential strategy for exploiting novel high-performance ceramic materials.

## 1. Introduction

Dielectric materials have received great attention for energy storage applications in microwaves, pulse power systems, electric vehicles, and high-frequency inverters owing to their ultra-fast charge–discharge rate and better temperature stability than batteries, electrochemical capacitors, and dielectric polymers [1,2,3,4,5,6]. However, dielectric capacitors have a lower energy storage density and efficiency than batteries and polymers because of their low dielectric breakdown strength (BDS), limiting their practical applications. Therefore, novel high-performance dielectric capacitors with high energy storage density and efficiency are needed for energy storage capacitor applications.

In general, the recoverable energy density (*W_rec_*) and energy efficiency (*η*) of a dielectric capacitor can be calculated from the polarization (*P*)–electric field (*E*) loop using the following equations [6,7,8,9,10]:

(1) $$W_{rec}=\int_{P_{r}}^{P_{max}}E\;dP$$

(2) $$\eta =\frac{W_{rec}}{W_{rec}+W_{loss}}$$

where *P_max_* is the maximum polarization, *P_r_* is the remnant polarization, and *W_loss_* is the energy loss. According to the above equations, the energy storage performance can be significantly improved by enhancing the difference between *P_r_* and *P_max_* (*ΔP*). The BDS is also an important parameter for energy storage, which means a higher BDS is required for a high *W_rec_*.

Dielectric materials, such as linear dielectric (LDE), ferroelectric (FE), anti-ferroelectric (AFE), and relaxor ferroelectric (RFE) materials, have been extensively used in energy storage capacitors [5,6]. Among these materials, the RFE materials (for example, Bi_0.5_Na_0.5_TiO_3_ (BNT)-, BaTiO_3_ (BT)-, and BiFeO_3_ (BFO)-composition-based materials) show high maximum polarization, small remnant polarization, and slim *P-E* loops due to the creation of polar nanoregions (PNRs). Additionally, they mostly display highly dynamic short-range FE orders. Nevertheless, the PNRs can transform into long-range FE orders with the increasing electric field, leading to high maximum polarization. After the field is removed, the induced FE orders can simply convert into PNRs, resulting in a small remnant polarization [11,12,13,14]. Thus, the RFEs typically exhibit high *W_rec_* and *η*. Therefore, lead-based RFEs have been extensively reported for applications in energy storage capacitors [15,16]. However, the toxicity of lead poses a risk to both human health and the environment, which has encouraged the development of alternative lead-free ceramic materials. Over the past few years, lead-free RFEs, including BT [17,18,19,20,21]-, BNT [3,22,23,24,25,26,27]-, NaNbO_3_ (NN)/K_0.5_Na_0.5_NbO_3_ (KNN) [28,29]-, and AgNbO_3_ (AN) [30,31]-based materials, have been discovered and used in energy storage capacitor applications.

BNT-based materials display a strong ferroelectric response due to their large-electric-field-induced polarization and strain [32,33]. Hybridization between the Bi^3+^ 6s^2^ and oxygen 2p orbitals can result in weak Bi-O covalency with off-center Bi^3+^, enabling substantial lattice relaxation and generating massive saturated polarization with high remnant polarization [34]. Additionally, the creation of PNRs is assisted by the local random fields caused by valency differences and chemical inhomogeneity [35,36]. In addition, the relaxor characteristics of the material can be enhanced by adding another phase (for example, tetragonal/orthorhombic) and modifying it with a suitable dopant/binary/ternary composition, enabling slim hysteresis loops [37]. Among these lead-free ceramics, the BNT-BT binary composition is a very popular system; it has a morphotropic phase boundary (MPB) between a rhombohedral and a tetragonal phase and also has a high maximum polarization; these features are responsible for higher energy storage density and efficiency [38]. Interestingly, the incorporation of SrTiO_3_ (ST) into the BNT-BT system plays a significant role in enhancing dielectric and ferroelectric properties [26,39,40].

In this paper, we improved the energy storage performance in BNT-BT ceramics with the incorporation of SBT by combining relaxor and antiferroelectric properties. Relaxor ferroelectric materials exhibit a heterogeneous polar state at the nanoscale, and long-range domains are destroyed due to the cation order–disorder and random fields, resulting in a slim *P–E* loop [41,42,43]. The BT-BNT combination exhibits an AFE/FE response with relaxor characteristics, which makes them it interesting for energy storage applications [44,45]. Sr_0.7_Bi_0.2_TiO_3_ (SBT) is a relaxor ferroelectric with a small coercive field, small remnant polarization, and diffused dielectric maxima over a wide temperature range. It was utilized to make a solid solution with BNT-BT to achieve a relaxor nature [46]. Combining relaxor and antiferroelectric properties with a fine grain size by the incorporation of SBT into BNT-BT enables an enhanced maximum polarization and a minimized *P-E* loop, leading to improved breakdown strength. An improvement in recoverable energy density of 1.02 J/cm^3^ and a high energy efficiency of 75.98% at a BDS of 89 kV/cm are achieved using 0.725 [0.94BNT-0.06BT]-0.275 SBT ceramics.

## 2. Materials and Methods

A conventional solid-state reaction method was used to prepare (1−*x*) [0.94 Bi_0.5_Na_00.5_TiO_3_– 0.06BaTiO_3_]– *x* Sr_0.7_Bi_0.2_TiO_3_ (1−*x* BNT-BT- *x* SBT; *x* = 0, 0.20, 0.225, 0.25, 0.275 and 0.30) ceramic powders. Raw materials were used, including Bi_2_O_3_, Na_2_CO_3_, BaCO_3_, TiO_2_, and SrCO_3_. They weighed as per a stoichiometric ratio and ball-milled for 24 h with ZrO_2_ balls in an ethanol solution. Further, BNT, BT, and SBT slurries were dried at 120 °C and calcined individually at temperatures of 800 °C, 900 °C, and 950 °C for 2 h and 3 h, respectively, for the formation of their phases. These calcined BNT, BT and SBT powders were well mixed and ball-milled again for 12 h for the preparation of a BNT-BT-SBT composition. Furthermore, 5 wt.% of polyvinyl alcohol was added to the obtained powders before the product was pressed at 10 MPa into disks with 10 mm diameters and a thickness of about 0.5 mm. These pellets were sintered at 1100 °C for 3 h with a heating and cooling rate of 5 °C/min. Lastly, Ag paste was applied on the surface of sintered samples to measure electrical characterizations.

The phase evolution of the BNT-BT-SBT ceramic samples was investigated using an X-ray Diffractometer (Rigaku/Ultima IV) with a Cu-Kα wavelength of 1.5406 Å and sweeping rate of 0.02039 degrees per minute at a voltage of 40 kV and current of 30 mA. Raman spectra were measured by a Raman spectrometer (JASCO, NRS-5100) with a laser wavelength of 532 nm. Surface morphology and elemental composition were investigated by a scanning electron microscope (SEM) (TESCAN/VEGA II LSU) equipped with an energy-dispersive spectrometer (EDS). Temperature- and frequency-dependent dielectric properties were assessed from room temperature (RT) to 400 °C and 100 Hz–1000 kHz using an impedance analyzer (Hewlett Packard, 4284A). *P-E* hysteresis loops were performed from RT to 200 °C using a ferroelectric tester (Aix ACCT Systems GmbH, TF Analyzer 2000).

## 3. Results and Discussion

**Figure 1:**
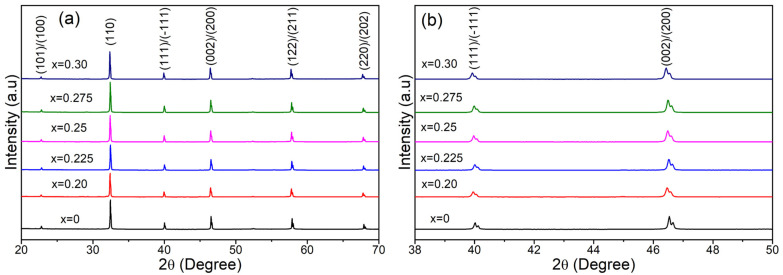
(**a**) X-ray diffraction patterns of the (1−*x*) BNT-BT- *x* SBT ceramic samples for *x* = 0–0.30 in the 2θ range of 20°–70°, and (**b**) enlarged XRD patterns in the range of 38°–50°.

Figure 1 shows the X-ray diffraction (XRD) patterns of (1−*x*) BNT-BT- *x* SBT ceramic samples for *x* = 0–0.30. The XRD peaks clearly indicate that all the samples of BNT-BT-SBT display the formation of rhombohedral (R) and tetragonal (T) phases, demonstrating that the SBT diffused into BNT-BT lattices to form a homogeneous solid solution. It is well known that the (1−*x*) BNT- *x* BT system possesses rhombohedral (Joint Committee on Powder Diffraction Standards (JCPDS) card No. 36-0340, 46-0001, Crystallography Open Database (COD) No. 2103295 for Bi_0.5_Na_0.5_TiO_3_) and tetragonal crystal structures (JCPDS card No. 89-1428, COD No. 1507756 and 2100858 for BaTiO_3_) at MPB for *x* = 0.06 [47,48,49,50,51,52,53,54]. The coexistence of both rhombohedral and tetragonal phases in (1−*x*) BNT-BT- *x* SBT ceramic samples for *x* = 0–0.30 was confirmed by the splitting of the (111)/(−111) and (002)/(200) peaks at 2θ about 40° and 46°, respectively, as shown in Figure 1b. However, no significant change in R and T phases was observed with the incorporation of the SBT composition. These results are in good agreement with the earlier reports on BNT-BT-based ceramic compositions [54,55]. In addition, these diffraction peaks shifted slightly to the lower-angle side with the addition of SBT into BNT-BT, indicating increased lattice parameters. The increased lattice parameters were responsible for Sr^2+^ (1.44 Å) having a larger ionic radius than Bi^3+^ (1.36 Å), Na^+^ (1.39 Å), and Ba^2+^ (1.35 Å) at the *A*-site [56,57,58].

**Figure 2:**
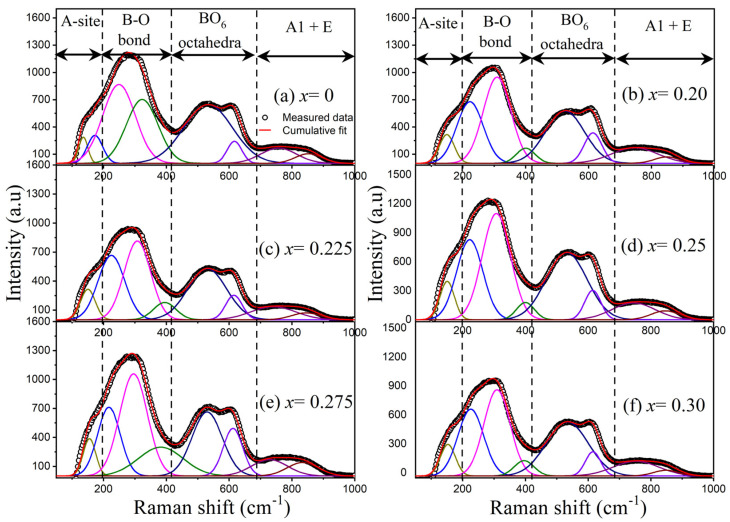
Raman spectra along with spectral deconvolution of (1−*x*) BNT-BT- *x* SBT ceramic samples for (**a**) x = 0, (**b**) x = 0.20, (**c**) x = 0.225, (**d**) x = 0.25, (**e**) x = 0.275, and (**f**) x = 0.30.

Figure 2 shows the Raman spectra of BNT-BT-SBT ceramic samples with spectral de-convolution. The Raman spectra of all the sintered samples are similar to those in the earlier reports on BNT-based ceramics [59,60]. The Raman modes separated into four regions, as illustrated at the top of Figure 2. (i) The Raman modes below 200 cm^−1^ were associated with the *A*-site (Bi-O, Na-O, Ba-O, and Sr-O) vibrations, (ii) the Raman modes between 200 and 420 cm^−1^ related to the B–O (Ti-O) vibrations, (iii) the modes between 420 and 690 cm^−1^ were associated with the BO_6_ (TiO_6_)–octahedra vibrations, and (iv) the Raman modes above 690 cm^−1^ corresponded to the *A1* and *E* overlapping modes [59]. The modes at 135–174 cm^−1^ were shifted to the higher wavenumbers of 159–218 cm^−1^ with SBT, related to the vibrations of *A*-site due to the *A*-site disorder. This disorder is produced by the addition of SBT (Bi^3+^ and Sr^2+^) into the BNT-BT (Bi^3+^, Na^+^, and Ba^2+^) system [61]. Additionally, a significant shift occurred at 312 and 530 cm^−1^ to lower wavenumbers of 296 and 524 cm^−1^ with the addition of SBT, which was attributed to the enhancement of *B*-site disorder in the BNT-BT-SBT [57]. Besides, these Raman active modes became broadened, indicating the disturbance of the long-range FE order and the creation of highly dynamic polar nanoregions and improving the relaxor characteristics of BNT-BT-SBT ceramics [62]. These findings are in good agreement with the X-ray diffraction, dielectric and ferroelectric properties.

**Figure 3:**
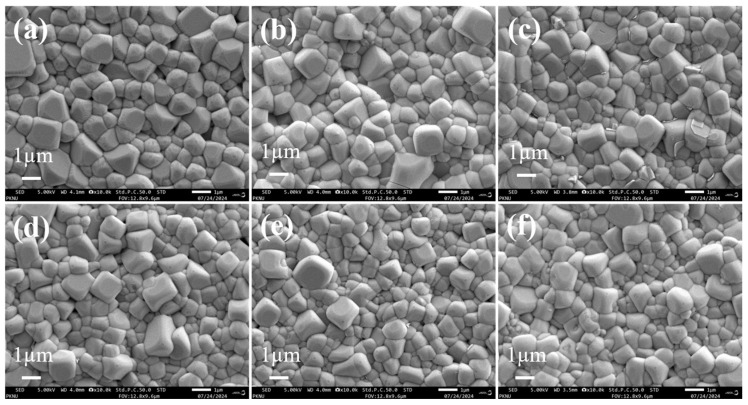
SEM images of (1−*x*) BNT-BT- *x* SBT ceramic samples for (**a**) *x* = 0, (**b**) *x* = 0.20, (**c**) *x* = 0.225, (**d**) *x* = 0.25, (**e**) *x* = 0.275, and (**f**) *x* = 0.30.

**Figure 4:**
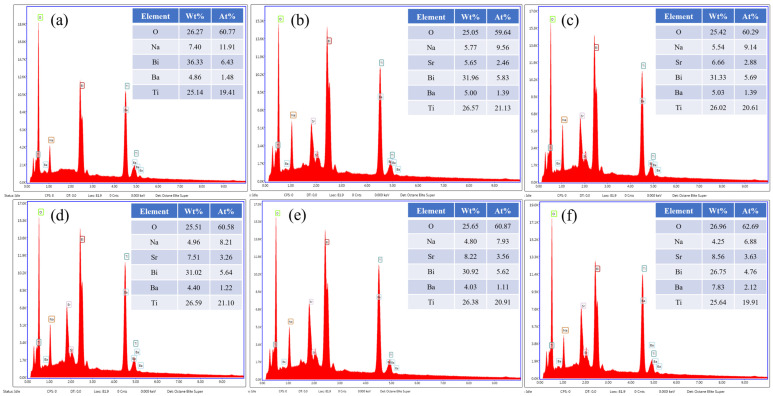
EDS spectra for the elemental analysis of (1−*x*) BNT-BT- *x* SBT ceramics: (**a**) *x* = 0, (**b**) *x* = 0.20, (**c**) *x* = 0.225, (**d**) *x* = 0.25, (**e**) *x* = 0.275, and (**f**) *x* = 0.30.

Figure 3 shows SEM images of the surface of BNT-BT-SBT ceramic samples. All the sintered ceramic samples show rectangular-shaped grains, which are uniformly distributed. Figure 3a clearly shows that the *x* = 0 sample displays a low-density microstructure, which gradually improves with the incorporation of SBT. The composition at higher concentrations shows a more compact and denser microstructure with smaller grains than other samples of (1-*x*) BNT-BT- *x* SBT, where *x* = 0.275 and *x* = 0.30. In order to prove that all the sintered ceramic samples have uniform microstructures and are highly dense, the density of BNT-BT-SBT ceramics was estimated using the Archimedes principle. The density was found to be enhanced from 5.62 ± 0.24 g/cm^3^ to 5.81 ± 0.28 g/cm^3^ with SBT from *x* = 0 to 0.30. The estimated relative density of BNT-BT-SBT was found to be 95.64% to 98.85% of the theoretical density [63], proving that these ceramic samples are highly dense and have homogeneous microstructures. Moreover, the average grain size of BNT-BT-SBT ceramic samples was calculated using Image-J 1.31 software using the linear intercept method. The average grain size was found to be 1.30 ± 0.41 µm for the *x* = 0 sample (pure BNT-BT), and further; it was slightly reduced to 0.81 ± 0.26 µm with the addition of SBT (*x* = 0.30). The reduction in grain size was ascribed to the larger radii of Sr^2+^ (1.44 Å) than Bi^3+^ (1.36 Å), Na^+^ (1.39 Å), and Ba^2+^ (1.35 Å) at the A-site, which enhanced the lattice strain energy (ΔG_strain_) [64,65]. The SEM results suggest that the sample with a fine grain size and a homogeneity and dense microstructure can sustain higher voltages, leading to improved BDS and energy storage properties [66,67]. Further, energy-dispersive X-ray spectroscopy (EDS) was performed to analyze the elemental composition of BNT-BT-SBT ceramics for *x* = 0–0.30. As seen in Figure 4, we assessed every element with the required composition and determined its atomic ratio. Moreover, A-site (Bi^3+^, Na^1+^, Ba^2+^, and Sr^2+^), B-site (Ti^4+^) and oxygen atomic and molecular proportions were nearly in line with the stoichiometric ratio of the BNT-BT-SBT compositions (Table 1). It is noticed that there was a small reduction in the ratio of Bi and Na elements due to their volatile nature during high sintering temperatures [68]. 

**Figure 5:**
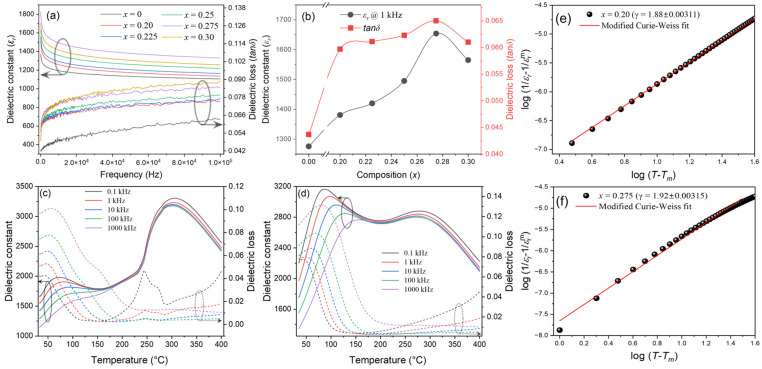
(**a**) Frequency variation in dielectric constant and dielectric loss of (1−*x*) BNT-BT- *x* SBT ceramic capacitors for *x* = 0–0.30. (**b**) Composition versus dielectric constant and dielectric loss. (**c**,**d**) Temperature dependency of dielectric constant and dielectric loss. (**e**,**f**) represent $log\left(T-T_{m}\right)$ versus log1εr−1εrm of (1-*x*) BNT-BT- *x* SBT ceramic for *x* = 0.20 and 0.275 at 1 kHz.

Figure 5a illustrates dielectric constant (*ε_r_*) and dielectric loss (*tanδ*) as functions of frequency for BNT-BT-SBT ceramics measured at RT from 100 Hz to 100 kHz. The sample *x* = 0 (BNT-BT) showed a lower *ε_r_* of 1276 and lower *tanδ* of 0.043 at 1 kHz as compared to pure BNT ceramics (*ε_r_* = 692 and *tanδ* = 0.045 at 1 kHz) [57,69]. The *ε_r_* and *tanδ* values were gradually enhanced to 1654 and 0.061 with the incorporation of SBT into BNT-BT for the *x* = 0.275 composition (as shown in Figure 5b). The improvement of the dielectric properties was attributed to the substitution of SBT into BNT-BT, a dense and homogeneous microstructure. Figure 5c,d show the temperature variation in *ε_r_* and *tanδ* of BNT-BT-SBT ceramics for lower and optimized compositions of *x* = 0.20 and 0.275, which are measured at different frequencies of 0.1, 1, 10, 100, 1000 kHz. It is noticed that the dielectric maximum temperature (*T_m_*) was shifted to a lower temperature with the addition of SBT for the composition of *x* = 0.275 than *x* = 0.20, as can be seen in Figure 5c,d. The addition of SBT into BNT-BT can disrupt the long-range FE order, and lower the dielectric maximum temperature. As a result, PNRs form owing to the difference in ionic radius at the *A*-site of BNT-BT-SBT ceramics. Moreover, the dielectric maximum temperature peaks are shifted to higher temperatures, and gradually become diffuse with frequency, which indicates the typical relaxor ferroelectric behavior [70,71]. The degree of the relaxor behavior of these samples was calculated using a modified version of the Curie–Weiss law [56,72]:

(3) 1εr−1εrm=T−TmγC

where $\varepsilon_{r}^{m}$ is maximum dielectric constant at a maximum temperature *T_m_*, *T* is absolute temperature, *C* is Curie constant, and *γ* is degree of relaxation. The value of *γ* is 1 for normal ferroelectrics and in between 1 and 2 for relaxor ferroelectrics [72]. Figure 5e,f display the log–log plots of (1/$\varepsilon_{r}\;-$ 1/$\varepsilon_{r}^{m}$) vs. (*T* − *T_m_*) of BNT-BT-SBT ceramics for the compositions of *x* = 0.20 and 0.275, which were performed at 1 kHz. The *γ* value slightly enhanced from 1.88 ± 0.00311 to 1.92 ± 0.00315, confirming the improved relaxor properties with the incorporation of SBT, resulting in high energy storage density and energy efficiency. These results are in good agreement with earlier reports [1,2,3].

**Figure 6:**
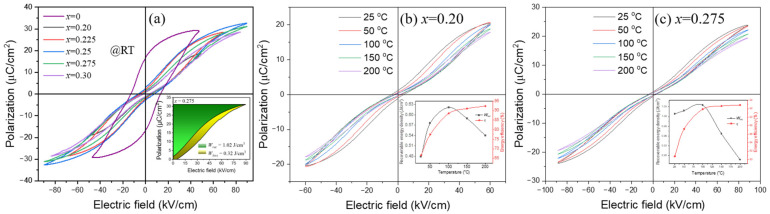
(**a**) RT bipolar *P-E* hysteresis loops of (1−*x*) BNT-BT- *x* SBT ceramic capacitors for all compositions from *x* = 0 to 0.30. The inset of Figure 6a shows the unipolar *P-E* loop with energy storage density and energy loss for the optimized sample (*x* = 0.275). (**b**,**c**) bipolar *P-E* hysteresis loops. Insets of (**b**,**c**) show *W_rec_* and *η* at different temperatures for *x* = 0.20 to 0.275.

Figure 6 shows the RT *P-E* loops of BNT-BT-SBT ceramic capacitors measured at 10 Hz. The BNT-BT-SBT sample (*x* = 0) displays typical FE characteristics. It shows a *P_r_* of 17.06 µC/cm^2^, *P_max_* of 29.29 µC/cm^2^, and coercive field (*E_c_*) of 13.03 kV/cm. These values initially decreased for *x* = 0.20 and further increased with SBT, whereas the *E_BD_* increased from 48.75 kV/cm to 89.73 kV/cm from *x* = 0 to 0.275, which provides a high energy storage density (inset of Figure 6a). The *P-E* loops are slim as compared with the ferroelectric sample with a composition of *x* = 0; their shape is actually a pinched ferroelectric loop. The “pinched” hysteresis loop in ferroelectric materials can result from two key mechanisms: defect pinning or intrinsic phase transitions. In the first case, defects like impurities or grain boundaries pin domain walls, preventing full polarization switching and creating a pinched loop. However, in defect-free systems, pinched loops can also arise from intermediate modulated phases that combine FE/AFE and RFE orders [73,74]. In this work, these phases form in (1-*x*) BNT-BT- *x* SBT as the composition changes, leading to incomplete polarization switching. The material undergoes a transition between these modulated states by the application of an electric field, but the polarization does not completely revert to its original state after the field is removed. This results in a “pinched” loop due to the coexistence of AFE and RFE orders, preventing total polarization reversal. Therefore, the observed behavior can be understood by intrinsic phase transitions.

Figure 6b,c show the bipolar *P-E* loops of BNT-BT-SBT ceramics measured at different temperatures and at 10 Hz. The energy storage density was increased with the temperature up to 100 °C, and further decreased due to diffusive FE to AFE transition (depolarization temperature). Dielectric and *P–E* hysteresis loop studies show that the *T_d_* is mostly dependent on BNT-BT-SBT concentrations. Moreover, the energy storage properties in terms of *W_rec_*, *W_loss_*, and *η* were estimated by Equations (1) and (2) from unipolar *P-E* loops and shown in Table 2. The *W_rec_* values gradually improved with the incorporation of SBT for *x* = 0 and 0.275. The sample *x* = 0.275 shows a high recoverable energy density of 1.02 J/cm^3^ at *E_BD_* of 89.73 kV/cm and an energy efficiency of 75.83%. The improvement in the energy storage performance is achieved by combining relaxor and antiferroelectric properties. The combining relaxor and antiferroelectric properties by incorporation of SBT into BNT-BT produced an enhanced maximum polarization and a slim *P-E* loop, leading to an improved BDS, an improved recoverable energy density of 1.02 J/cm^3^, and a high energy efficiency of 75.98% at a BDS of 89 kV/cm using 0.725 [0.94BNT-0.06BT]-0.275 SBT lead-free ceramics. The values of *W_rec_* and *η* of 0.725 (0.94 BNT-0.06 BT)-0.275 SBT ceramic capacitors are almost superior to, and comparable to, those of other lead-free ceramic materials, which are good candidates for energy storage capacitor applications [32,75,76,77,78,79,80]. 

## 4. Conclusions

A series of BNT-BT-SBT relaxor ferroelectric ceramics were fabricated using a conventional solid-state reaction method and we determined their energy storage properties by combining relaxor and antiferroelectric properties. XRD, Raman spectra, and SEM characterizations confirmed the formation of a rhombohedral–tetragonal phase, the presence of highly dynamic polar nanoregions, and reductions in grain size with homogeneous and dense microstructures. The dielectric and ferroelectric properties were enhanced. A high dielectric constant, low dielectric loss, and low remnant polarization were found with the incorporation of SBT into BNT-BT for *x* = 0.275 composition, which was beneficial for improving energy storage performance. Combining relaxor and antiferroelectric properties with fine grain size contributes a large breakdown strength. There is also large difference between *P_max_* and *P_r_* with the substitution of SBT, leading to high energy storage density and a high energy efficiency being obtained for the 0.725 [0.94BNT-0.06BT]-0.275 SBT composition. Our results demonstrate that combining relaxor and antiferroelectric properties is an effective approach and that BNT-BT-SBT ceramics are promising for energy storage capacitor applications.

## Figures and Tables

**Table 1:** Elemental composition of (1−*x*) BNT-BT- *x* SBT ceramic samples for *x* = 0–0.30.

Samples	Composition	
	Measured Atomic %	Theoretical Atomic %
X = 0	O = 60.77, Na = 11.91, Bi = 6.43, Ba = 1.48 and Ti = 19.41	A-site (Na = 9.4, Bi = 9.4 and Ba = 1.2), B-site (Ti = 20) and O = 60
X = 0.20	O = 59.64, Na = 9.56, Sr = 2.46, Bi = 5.83, Ba = 1.39 and Ti = 21.13	A-site (Na = 7.52, Bi =8.32, Ba = 0.96 and Sr = 2.24), B-site (Ti = 20) and O = 60
X = 0.225	O = 60.29, Na = 9.14, Sr = 2.88, Bi = 5.69, Ba = 1.39 and Ti = 20.61	A-site (Na = 7.285, Bi = 8.185, Ba = 0.93 and Sr = 2.52), B-site (Ti = 20) and O = 60
X = 0.25	O = 60.58, Na = 8.21, Sr = 3.26, Bi = 5.64, Ba = 1.22 and Ti = 21.10	A-site (Na = 7.05, Bi = 8.05, Ba = 0.9 and Sr = 2.8), B-site (Ti = 20) and O = 60
X = 0.275	O = 60.87, Na = 7.93, Sr = 3.56, Bi = 5.62, Ba = 1.11 and Ti = 20.91	A-site (Na = 6.815, Bi = 7.915, Ba = 0.87 and Sr = 3.08), B-site (Ti = 20) and O = 60
X = 0.30	O = 62.69, Na = 6.88, Sr = 3.63, Bi = 4.76, Ba = 2.12 and Ti = 19.91	A-site (Na = 6.58, Bi = 7.78, Ba = 0.84 and Sr = 3.36), B-site (Ti = 20) and O = 60

**Table 2:** The values of ferroelectric (*P_r_*, *P_max_*, *E_c_,* and *E_BD_*) and energy storage properties (*W_rec_*, *W_loss,_* and *η*) of (1-*x*) BNT-BT- *x* SBT ceramic capacitors for *x* = 0–0.30.

Composition	*P_r_* (µC/cm^2^)	*P_max_* (µC/cm^2^)	*E_c_* (kV/cm)	*E_BD_* (kV/cm)	*W_rec_* (J/cm^3^)	*W_rec_* (J/cm^3^)	*η* (%)
*x* = 0	17.06	29.29	13.03	43.99	0.16	0.80	17.45
*x* = 0.20	0.79	18.76	2.19	48.75	0.39	0.14	73.58
*x* = 0.225	2.09	28.38	4.77	68.99	0.68	0.37	64.76
*x* = 0.25	2.95	32.82	6.47	89.38	0.93	0.50	65.03
*x* = 0.275	1.39	31.39	2.97	89.73	1.02	0.32	75.83
*x* = 0.30	1.00	28.54	2.33	83.53	0.96	0.22	81.35

## Data Availability

Data sharing is not applicable.
